# WHO Environmental Noise Guidelines for the European Region: A Systematic Review on Environmental Noise and Adverse Birth Outcomes

**DOI:** 10.3390/ijerph14101252

**Published:** 2017-10-19

**Authors:** Mark J. Nieuwenhuijsen, Gordana Ristovska, Payam Dadvand

**Affiliations:** 1 ISGlobal, Centre for Research in Environmental Epidemiology (CREAL), 08003 Barcelona, Spain; payam.dadvand@isglobal.org; 2 Universitat Pompeu Fabra (UPF), 08002 Barcelona, Spain; 3 CIBER Epidemiologia y Salud Publica (CIBERESP), 28029 Madrid, Spain; 4 Institute of Public Health of Republic of Macedonia, Skopje 1000, Macedonia; ristovskagordana@gmail.com; 5 Medical Faculty, University Sts Ciril and Methodius, Skopje 1000, Macedonia

**Keywords:** noise, gestation, pregnancy, prematurity, congenital anomaly, congenital abnormality, quality of evidence

## Abstract

*Introduction*: Three recent systematic reviews suggested a relationship between noise exposure and adverse birth outcomes. The aim of this review was to evaluate the evidence for the World Health Organization (WHO) noise guidelines and conduct an updated systematic review of environmental noise, specifically aircraft and road traffic noise and birth outcomes, such as preterm birth, low birth weight, being small for gestational age and congenital malformations. *Materials and methods*: We reviewed again all the papers on environmental noise and birth outcomes included in the previous three systematic reviews and conducted a systematic search on noise and birth outcomes to update previous reviews. Web of Science, PubMed and Embase electronic databases were searched for papers published between June 2014 (end date of previous systematic review) and December 2016 using a list of specific search terms. Studies were also screened in the reference list of relevant reviews/articles. Further inclusion and exclusion criteria for the studies provided by the WHO expert group were applied. Risk of bias was assessed according to criteria from the Newcastle-Ottawa quality assessment scale for case-control and cohort studies. Finally, we applied the GRADE principles to our systematic review in a reproducible and appropriate way for judgment about quality of evidence. *Results*: In total, 14 studies are included in this review, six studies on aircraft noise and birth outcomes, five studies (two with more or less the same population) on road traffic noise and birth outcomes and three related studies on total ambient noise that is likely to be mostly traffic noise that met the criteria. The number of studies on environmental noise and birth outcomes is small and the quality of evidence generally ranges from very low to low, particularly in case of the older studies. The quality is better for the more recent traffic noise and birth outcomes studies. As there were too few studies, we did not conduct meta-analyses. *Discussion*: This systematic review is supported by previous systematic reviews and meta-analyses that suggested that there may be some suggestive evidence for an association between environmental noise exposure and birth outcomes, although they pointed more generally to a stronger role of occupational noise exposure, which tends to be higher and last longer. Very strict criteria for inclusion and exclusion of studies, performance of quality assessment for risk of bias, and finally applying GRADE principles for judgment of quality of evidence are the strengths of this review. Conclusions: We found evidence of very low quality for associations between aircraft noise and preterm birth, low birth weight and congenital anomalies, and low quality evidence for an association between road traffic noise and low birth weight, preterm birth and small for gestational age. Further high quality studies are required to establish such associations. Future studies are recommended to apply robust exposure assessment methods (e.g., modeled or measured noise levels at bedroom façade), disentangle associations for different sources of noise as well as daytime and nighttime noise, evaluate the impacts of noise evens (that stand out of the noise background), and control the analyses for confounding factors, such as socioeconomic status, lifestyle factors and other environmental factors, especially air pollution.

## 1. Introduction

Environmental noise is related to a range of adverse health outcomes, such as impaired cognitive function in children, sleep disturbance, and cardiovascular diseases [1]. A number of studies have investigated the relationship between environmental and occupational noise and birth outcomes including low birth weight (LBW), small for gestational age (SGA), preterm birth (PTB), spontaneous abortion, and congenital malformations, which were reviewed in a few systematic reviews [2,3,4].

The World Health Organization (WHO) defines PTB as a gestational age at birth of less than 37 completed gestational weeks, and LBW as birth weight of less than 2500 g irrespective of gestational age. LBW has been associated with not only poorer health and development in children, but also with adverse health outcomes in later life. It, for example, is a predictor infant mortality as well as impaired educational achievement and increased risk of enhanced risk of noncommunicable diseases (NCDs) such as of ischemic heart disease, chronic hypertension, and insulin resistance/metabolic syndrome in adults. The World Health Assembly has endorsed targets to reduce LBW prevalence by 30% by 2025 [3,5,6]. Similarly, PTB is a public health issue of global significance, which may result in mortality during the perinatal period or may lead to major health and financial consequences due to lifelong impacts. Preterm birth complications are the leading cause of death among children under 5 years of age, responsible for nearly 1 million deaths in 2015. Even though several risk factors for preterm birth have been identified, prevention efforts have failed to halt the increasing rates of preterm birth [5,6].

Epidemiological studies have identified air pollution as an emerging potential risk factor for PTB and LBW, where inflammation has important role, through direct stimulation of inflammatory cells or mediated by oxidative stress [7]. Occupational studies identified several occupational and non-occupational factors that have influence on birth outcomes—occupational noise exposure, exposure to chemicals, high ambient temperature—and usually persons who were exposed to noise were exposed to other occupational factors. Mother’s age, weight and height, weight gain during pregnancy, smoking, education, race and socioeconomic status, gravidity and parity, and chronic diseases are important factors to be consider while evaluating the effects of environmental factors on pregnancy outcomes [3,4].

Hohmann et al. (2013) conducted a systematic review to examine associations between chronic noise exposure during pregnancy or childhood and health outcomes in early and late childhood. They included 12 studies on pregnancy outcomes and rated evidence levels (3 to 2++) according to the Scottish Intercollegiate Guidelines Network and concluded that chronic noise exposure during pregnancy was not associated with birth weight, preterm birth, congenital malformations, perinatal and neonatal death based on six cohort, four case–control, and two cross-sectional studies (highest evidence level 2+). They also reported that the studies included in the systematic review showed a high variation in study design, outcome, exposure and confounder assessments [2].

Ristovska et al. (2014) conducted another systematic review of available evidence on the association between environmental noise exposure and pregnancy outcomes such as LBW, PTB, spontaneous abortion, and congenital malformations. They included nine studies in their review, encompassing four case-control, two surveys, one cross-sectional study, one prospective study and one population-based cohort study. Objective noise measurements were used in eight of these studies. Most studies examined aircraft noise exposure and its influence on low birth weight. According to their quality assessment score, six studies were assessed as providing strong evidence and three studies provided a moderate evidence score. They concluded that there is some suggestive evidence of adverse associations with noise from both occupational and epidemiological studies, especially for low birth weight [3].

Dzhambov et al. (2014) conducted a systematic review and meta-analyses on 29 studies related to noise exposure and pregnancy complications and outcomes. They also assessed the quality of evidence and included both occupational and environmental studies of noise, but did not separate in the analyses the type of exposure, which is a limitation for this review. Women exposed to high noise levels (in most of the studies ≥80 dB) during pregnancy were at a significantly higher risk for having small-for-gestational-age newborn (RR = 1.19, 95% CI: 1.03, 1.38), gestational hypertension (RR = 1.27, 95% CI: 1.03, 1.58) and offspring with congenital malformations (RR = 1.47, 95% CI: 1.21, 1.79). The effects were not statistically significant for preeclampsia, perinatal death, spontaneous abortion and preterm birth [4].

A change of approach is vital, moving from addressing individual risk factors of pregnant women to addressing co-occurring groups of factors with the whole family, household and community around the women most at risk. First priority goal of Health Strategy 2020 is investment in life course approach and empower people, especially for children to have good start in life, learn better and have more productive lives [8].

The area of research on the association between noise exposure and birth outcomes is rapidly evolving. The translation of evidence in this area into policy, therefore, requires regular updates of the synthesized available evidence. Here we provide an update of the evidence for the WHO noise guidelines and conduct an updated systematic review of environmental noise, specifically aircraft and road traffic noise and birth outcomes.

## 2. Methods

We reviewed again all the papers included in the three aforementioned systematic reviews and conducted a systematic search on noise and reproductive outcomes. After careful reading of full papers included in the previous reviews we found that only 8 papers satisfied inclusion criteria, developed for this review (Table 1).

**Figure 1:**
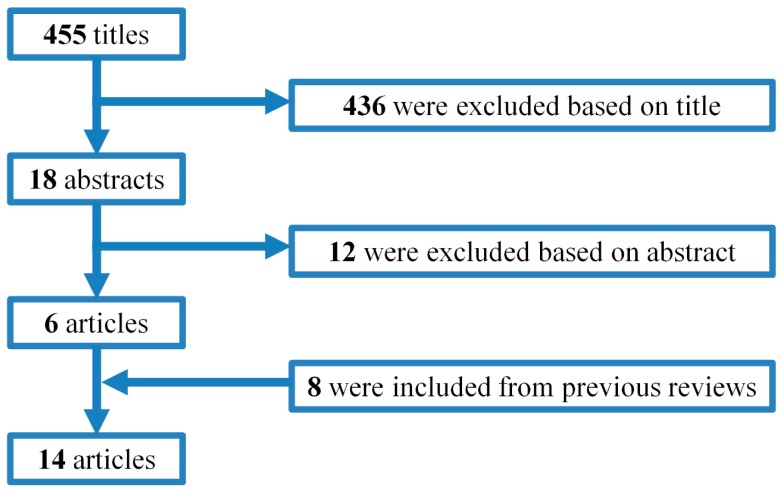
Study identification and selection.

Web of Science, PubMed and Embase electronic databases were searched for papers published between June 2014 (end date of previous systematic review) and December 2016. Studies were also screened in the reference list of relevant reviews/articles. In addition, hand searching was used for acoustical conference proceedings. No language restriction was applied. The following search terms were used: noise AND health AND perinatal OR prenatal OR labor OR birth OR malformation OR gestation OR preterm OR fetus OR pregnancy. We found 455 titles of the studies, but after careful reading of the abstracts and full papers we decided that only 6 studies met the inclusion criteria (Figure 1).

Studies were included if: (a) noise exposure assessment was performed with noise measurements or noise mapping, (b) the source of noise was environmental (road traffic, railway or aircraft noise), (c) the study investigated the following reproductive outcomes: birth weight/gestational length/preterm birth/prematurity/reproductive health/congenital malformations/fetal growth retardation/small-for-gestational-age infant/spontaneous abortion, (d) the above health outcomes occurred during pregnancy or delivery up to 4 weeks after birth and (e) the paper examined a relationship between the above health outcomes and noise exposure. Studies investigating health outcomes other than those listed in the inclusion criteria such as preeclampsia, hearing development, male reproductive function, or health outcomes that occurred after the 4th week of birth were not included in this review. Case studies or case reports, studies containing no original research and studies investigating different noise source such as neonatal intensive care unit (NICU) noise were excluded as were studies looking at distance from road only without other assessment of noise exposures.

We used data extraction sheets designed for the following entries: author, year of publication, country, study design, sample size, exposure assessment (indicators and range of exposure), outcome, confounding factors, potential for bias, effect size and quality score following an earlier systematic review by Ristovska et al. (2014). We used a quality assessment (QA) for epidemiological studies based on criteria from the Newcastle-Ottawa quality assessment scale for case-control and cohort studies [9]. The criteria included were:

Publication type (0 = not peer reviewed, 1 = peer reviewed article),
Study design (1 = ecological, 2 = case control or cohort study, 3 = RCT, 0 = other),
Noise exposure assessment (3 = objective assessment with noise measurements or noise calculations),
Assessment of the birth outcomes (1 = subjective assessment by report of mother, 2 = objective e.g., from medical records),
Confounding factors (0 = no confounding factors considered, 1 = confounding factors considered but some key confounders omitted, 3 = careful consideration of confounders),
Statistics (0 = flaws in or inappropriate statistical testing or interpretation of statistical tests that may have affected results, 1 = appropriate statistical testing and interpretation of tests),
Bias (0 = other study design or conduct issues that may have led to bias, 1 = no other serious study flaws).

Based on this scale, the maximum total score can be 14. Studies with a score of ≥10 were assessed as at low risk of bias, studies with a score from 6 to 9 were assessed as at unclear risk of bias, and studies with a score ≤5 were assessed as at high risk of bias.

Finally, we applied the GRADE (Grading of Recommendations, Assessment, Development and Evaluations) principles to our systematic review in a reproducible and appropriate way for judgments about quality of evidence.

## 3. Results

In total, from the previous systematic reviews and new search we found 14 studies (Figure 1) including six studies on aircraft noise and birth outcomes (Table 2), five (two with more or less the same population) on road traffic noise and birth outcomes and three on total ambient noise (that, given the context, were likely to be mainly traffic noise) that met the criteria (Table 3). We did not exclude any studies from the evaluation.

## 4. Aircraft Noise

### 4.1. Birth Weight and Preterm Birth

Ando and Hattori (1973) reported that there was an increased incidence of low birth weight in babies from mothers exposed to aircraft noise. The mothers were divided into five subgroups of exposure in range of 74–90 dBA using the ECPNL (Equivalent Continuous Perceived Noise Level) indicator. The relative low birth weight rate was 3% lower for in the noisy area (above 75 dBA) compared to neighboring quiet cities, which were not exposed to jet aircraft flights. The relative low birth weight rate increased further to over 5% for both males and females when the planes started to fly more regularly over the affected areas [10].

In a small study involving 115 infant, s Schell (1981) examined the association between maternal exposure to aircraft noise and birth weight and gestational length. They performed a noise exposure assessment with measurements during airplane takeoff (range of 75–100 dBA). They collected birth weight and other data through personal interviews with the mothers. They reported a significant negative partial correlation between noise exposure and gestational length in female infants, after adjusting for maternal age, smoking, parity, socioeconomic status, and parental height and weight (r = −0.49, *p* < 0.001). They also found a weak non-statistically significant negative correlations between noise exposure and male birth weight and gestational length and with female birth weight [11].

In a study in the Netherlands, Knipschild et al. (1981) compared the rate of low birth weight in 498 infants whose mother lived in a noisy area near the Amsterdam airport with that of 404 infants from less noisy areas. Eighteen percent of babies were of low birth weight in areas exposed to Ldn < 65 dBA and 23% in areas exposed to Ldn 65–70 dBA. They adjusted their analyses for parent’s income, mother’s age, birth order, twinship and sex of the infant (but not for mother’s smoking) and found that after adjustment for family income, there was only an association among female babies [12].

In Japan, in a large study (160,460 birth records) Matsui et al. (2003) found a strong statistically significant dose-response relationship between aircraft noise exposure and low birth weight in the highest exposure group in the town of Kadena (adjusted odds ratio (OR) for low birth weight was 1.32, 95% confidence intervals (CI): 1.18, 1.48 and for preterm birth was 1.25, 95% CI: 1.09, 1.44, average noise levels 88 dB of WECPNL(weighted equivalent continuous perceived noise level). They adjusted their analyses for the gender of the baby, maternal age, birth order, occupation of householder, but not for smoking of the mothers, which may have resulted in some residual confounding [13].

### 4.2. Congenital Malformations

Near Los Angeles airport using an ecological study design, Jones and Tauscher (1978) reported a greater incidence of all congenital malformations combined among black babies in areas where the noise exposure was >90 dBA compared to those babies who were not exposed to aircraft noise (1185 vs. 737 per 100,000 births *p* < 0.02). For white babies of mothers who lived near the airport, there was an increased incidence of anencephaly and spina bifida [14].

Near Atlanta airport, Edmonds et al. (1979) reported on the incidence of congenital malformations combined (1745 cases) in two groups of infants whose mothers lived around the airport and were exposed to Ldn above 65 dB or below 65 dB. They did not report a statistically significant association [15].

Based on the above evidence the GRADE quality scores are provided in the Tables S1–S3 for aircraft noise and various outcomes. Given the available studies, the evidence for associations is graded as very low.

## 5. Road Traffic Noise

### Birth Weight and Preterm Birth

Wu et al. (1996) conducted detailed noise assessments during pregnancy in a cohort of 200 pregnant women using personal noise dosimeter performing noise measurements for 24 h, noise maps for residential areas of the participants; and self-reported exposure to loud music and using personal listening devices. The mean level and standard deviation of individual exposure Leq 24 h was 67.9 dBA, (52.4 dBA–86.8 dBA). The birth weight was obtained from medical records. They reported no statistically significant associations between personal noise exposure measured and low birth weight (*p* = 0.24), traffic noise exposure and low birth weight (*p* = 0.17), and using personal listening musical devices and birth weight (*p* = 0.34) [16].

In Vancouver, Canada, Gehring et al. (2014) examined the association between modeled residential road traffic and all transportation noise exposure and birth outcomes in nearly 70,000 administrative birth records. They focused on road traffic noise as railways and airports were minor contributors to overall community noise in this region. They reported a statistically significant negative association between road traffic exposure and term birth weight with mean difference = −19 g (95% CI = −23 to −15) per 6 dBA after controlling for various factors including income and education. Adjustment for air pollution exposure did not change the results. They reported similar sized negative associations for combined road, aircraft and railway noise. They also reported a statistically significant increased risk for small for gestational age OR = 1.10 (1.06–1.13), but not for preterm or very preterm birth. In joint noise-air pollution models, they reported independent associations between noise and air pollution exposure and small for gestational age [17].

In Barcelona, Spain (2001–2005), Dadvand et al. (2014) reported on a cohort study that was based on 6438 singleton term births. They obtained information on exposures to air pollution, noise, and heat using, temporally adjusted land-use regression models, annual averages of 24-h noise levels based on regulatory noise map of Barcelona, and average of satellite-derived land-surface temperature, respectively. They did not find any statistically significant association for noise, but did for air pollution and heat exposures [18].

In Vancouver, British Columbia, Canada, in the same cohort as Gehring et al. (2014), Hystad et al. (2014) [19] reported on the associations between residential greenness and birth outcomes in a cohort of 64,705 singleton births (from 1999–2002) and adjusted for noise. Reported noise effect estimates were more or less the same as the Gehring et al. (2014) study [17].

In Denmark, Hjortebjerg et al. (2016) investigated the associations between residential air pollution and traffic noise during pregnancy and newborn's size at birth. From a national birth cohort in Denmark they identified 75,166 live-born singletons born at term with information on the newborn’s size at birth. Residential address history from conception until birth was collected and air pollution (NO_2_ and NO_x_) and road traffic noise was modeled at all addresses. Associations between exposures and indicators of newborn’s size at birth: birth weight, placental weight and head and abdominal circumference were analyzed by linear and logistic regression, and adjusted for potential confounders. In mutually adjusted models they found that exposure to residential road traffic noise was weakly associated with reduced head circumference, whereas none of the other newborn's size indicators were associated with noise, neither before nor after adjustment for air pollution. This study indicates that traffic noise seems not to affect newborn's size at birth [20].

In Madrid three related studies examined the relationship between short term noise levels and adverse birth outcomes. Noise was measured using ambient monitoring stations that were probably capturing, to a large extent but not exclusively, traffic-related noise levels.

Arroyo et al. (2016a) conducted a ecological time-series study to assess the impact of air pollution (PM_2.5_, NO_2_ and O_3_), noise exposure, and ambient temperature on low birth weight and preterm birth in Madrid across the period 2001–2009. The mean Leqd value was 64.6 dB(A), with a daily maximum value of 69.0 dB(A), while the mean Leqn value was 59.4 dB(A), with a daily maximum value of 67.5 dB(A) noise values were exceeded on 45% of days and 100% of nights across the period analyzed. Regarding low birth weight, Leqd was the variable that had the most impact across the gestational period, displaying an association for exposure in weeks 3 (first trimester), 21 (second trimester) and 37 (third trimester) of pregnancy. Their results for preterm birth suggested that noise levels had an impact in two ways, namely: Leqd in week 21 (second trimester), midway through the pregnancy, coinciding with the explanatory lag for low birth weight; and Leqn, with a statistically significant association in week 36 (third trimester) of pregnancy. They concluded that special mention should be made of the effect of noise, not only because it acts continuously across the entire pregnancy, but also because it does so in an acute form in its role as a trigger of labor process. However, the study had serious limitations because they didn’t control confounding factors of mothers [21].

Arroyo et al. (2016) conducted ecological time-series analysis on the same study sample as mentioned above and same model for noise exposure assessment to assess the short-term impact of daily mean concentrations of air pollutants, noise and heat waves on preterm birth, classified as very preterm births and extremely preterm births. For diurnal noise exposure Leqd (Lag 0), they found an increased risk (relative risk (RR) of 1.07, 95% CI: 1.04, 1.10) for very preterm birth associated with significant attributable risk of 6.89, and for extremely very PB (RR of 1.28 (95% CI: 1.21, 1.36) with an attributable risk of 22.23. Confounding factors from maternal lifestyle and socioeconomic status were not included in the analysis [22].

Diaz et al. (2016) conducted an ecological time-series study to assess the impact of PM_2.5_, NO_2_ and O_3_ concentrations, measured noise levels at monitoring stations, and temperatures on LBW among non-premature infants over the period 2001–2009 based on the same study sample and measures of exposure as the aforementioned studies by Arroyo et al. Their analysis extended to infants having birth weights of 1500 g to 2500 g classified as very low birth weight (VLBW) and less than 1500 g classified as extremely low birth weight (ELBW). Environmental variables were lagged until 37 weeks with respect to the date of birth, and cross-correlation functions were used to identify explaining lags. Results were quantified using Poisson regression models. The relative risk of Leqd on low birth weight was 1.09 (95 CI: 0.99–1.19) (*p* < 0.1). Leqd, however, displayed an appreciably higher AR than that of PM, i.e., around 8%, though this was exclusively for the variable, low birth weight, and this increase in risk was significant at only *p* < 0.1 [23]. No association was reported for VLBW and ELBW. Based on the above evidence the GRADE quality scores are provided in the Tables S4–S6 for the road traffic and low birth weight, preterm birth, and small for gestational age. The quality for evidence for road traffic noise and low birth weight, preterm birth and small for gestational age was graded as low.

For other noise exposures no evidence is available.

## 6. Discussion

We found that the number of studies on environmental noise and birth outcomes is small and many of these studies have serious limitations such as not properly addressing confounding factors [21,22,23]. Earlier studies of the association between noise and pregnancy outcomes mainly dealt with aircraft noise exposure, while more recent studies were mostly focused on road and railway traffic noise exposure. More recent studies also tended to better address the combined impacts of noise with air pollutants and temperature. The quality of evidence was generally low, particularly in the case of the older studies. The quality of evidence is better for the more recent traffic-related noise and birth outcomes studies. Using GRADE principles we graded the quality of evidence for associations between aircraft noise and preterm birth, low birth weight and congenital anomalies as very low and the evidence for road traffic noise and low birth weight, preterm birth and small for gestational age as low quality. As there were too few studies with low risk of bias, we did not conduct meta-analyses. Our systematic review is supported by previous systematic reviews and meta-analyses that suggested that there may be some suggestive evidence for an association between environmental noise exposure and birth outcomes, although they pointed generally more to a stronger role of occupational noise exposure, which tends to be higher and last longer [2,3,4].

Dzhambov et al. (2014) concluded in their meta-analyses that women exposed to noise levels above 80 dBA during pregnancy are at a significantly higher risk for having small-for-gestational-age newborn and infant with congenital malformations. They found a 19% risk for small-for-gestational-age if the mother has been exposed to ≥80 dBA during pregnancy. All studies used a cut point of approximately 80 dBA noise exposure assessed either by specific question about the acoustic environment at work or by quantification by industrial hygienists. That mean the risk was calculated mainly on evidence obtained with studies related to occupational noise exposure, which are not included in our review [4].

Ristovska et al. (2014) in their review concluded that a small number of available studies were generally supportive of an association between noise exposure and adverse effects on low birth weight, but publication bias cannot be ruled out and some studies had limitations in design. The two largest studies found associations with LBW. One study from Japan [9] found significant risk for LBW for mothers exposed to aircraft noise above 85 dBA and another large population base cohort study from Canada [13] that found adverse effects of road traffic noise exposure and for all transportation noise associated with term birth weight and term very low birth weight [3].

Very strict criteria for inclusion and exclusion of studies, performance of quality assessment for risk of bias, and finally applying GRADE principles for judgment of quality of evidence are the strengths of this review. Some of the studies found significant risk for adverse birth outcomes, such as LBW, PTB or SGA, but according GRADE criteria for study limitations, inconsistency, directness, precision, publication bias, dose-response, magnitude of effect, confounding adjustment, they were assessed for quality of evidence as very low for aircraft noise exposure or quality of evidence as low for road traffic noise exposure (Tables S1–S6 in Supplementary Materials).

Although, the number of studies available on the impact of noise exposure on pregnancy outcomes is still small, the fact that we found six studies for the period from August 2014 to December 2016 could mean that this field of research is emerging. Investigation of combined effects of environmental factors is necessary, especially when the research is devoted to birth effects related to traffic pollution, or living in urban environment. Recently developed methods for exposure assessment based on traffic sources and modeling could provide great opportunity for further research work.

### 6.1. Biological Mechanism

Pregnancy is a physiological state characterized by increased hypothalamus-pituitary-adrenal (HPA) axis function and progressively increasing levels of serum concentrations of stress hormones including cortisol and adrenocorticotropic hormones (ACTH) after 12 weeks gestation. Corticotropin-releasing hormone (CRH), the principal regulator of the hypothalamic–pituitary–adrenal axis, has been identified in most female reproductive tissues including the uterus, the placenta, and the ovary. Placental CRH has been proposed to directly modulate the endocrine function of placental trophoblasts, including the production of estrogen, ACTH, and prostaglandin, and is involved in the timing of parturition. Remarkably the trajectory of CRH increase during pregnancy has been described to differ by ethnicity and sociodemographic factors. Stress hormones have inhibitory effects on female reproductive organs are responsible for inadequate levels of progesterone during pregnancy, subsequently resulting in preterm birth [3,4].

The possible biological mechanism were recently discussed by Dzhambov et al. (2014) and include a general stress response mechanism leading to neuroendocrine secretion [24,25,26] through activation of the amygdala, and some cortical limbic and hypothalamic centers [27], thereby affecting synaptic links in the reticular formation and mesencephalon, as well as emotional and cognitive pathways of perception through cortical and subcortical structures [28,29] finally leading to stimulation of the sympathetic-adrenal axis [30]. Stress generally triggers the release of neurohormones by the HPA axis, and thereby up-regulates key stress hormones such as CRH, ACTH and glucocorticoids (GCs) [22]. Furthermore, stress activates the sympathetic nervous system leading to increased secretion of catecholamines, a phenomenon that has received much less attention than the stress-triggered activation of the HPA axis. Blood pressure and uterine reactivity may be increased through stress-release of maternal catecholamine and thereby decreased placental function leading to hypoxia of the fetus [31]. Also maternal cortisol might pass through the placental barrier and interfere in the regulation of the fetal hypothalamic-pituitary-adrenal axis, or stimulate the placenta to secrete corticotropin releasing hormone [32]. Also noise energy is suggested to be able to affect the fetus directly [33]. Furthermore, neurotrophin nerve growth factor (NGF) which has a critical arbitrator role in stress responses and promotes “cross-talk” between neuronal and immune cells, could ultimately skew the immune response towards inflammation [34] which could be possible mechanism underlying the association between noise exposure and pregnancy outcomes.

Annoyance and sleep disturbance are among the most prevalent community response in a population exposed to environmental noise. General stress model is behind this reactions, potential mechanisms are emotional stress reactions due to perceived discomfort (indirect pathway), and non-conscious physiological stress from interactions between the central auditory system and other regions of the CNS (direct pathway). But for sleep disturbance direct pathway is dominant mechanism even at low noise levels. These effects have additional burden on stress response of pregnant women and individual noise sensibility has very important role [1,30].

**Figure 2:**
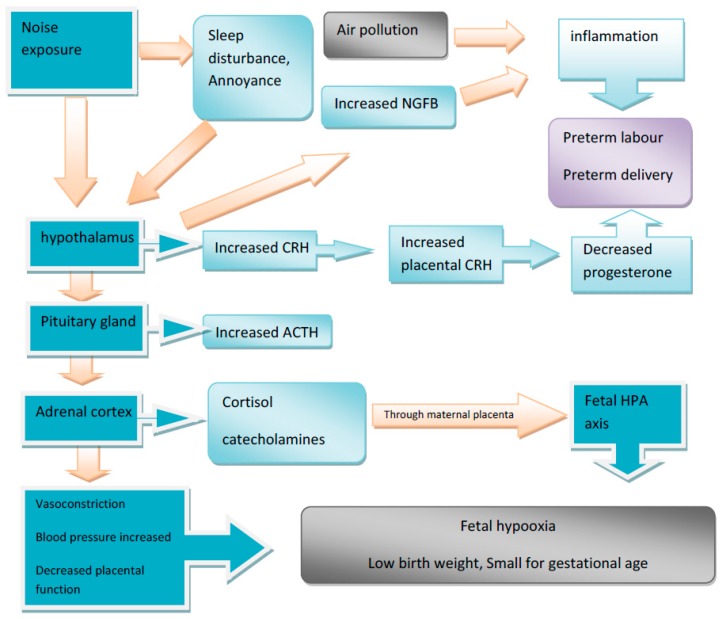
Graphic outline of possible biological mechanism for birth effects.

We prepared figure for pathway of possible biological mechanism for developing birth outcomes related to noise exposure (Figure 2).

### 6.2. Knowledge Gaps and Recommendations

Further studies are urgently needed on noise from different sources and pregnancy outcomes, given the suggestion that noise may affect pregnancy outcomes and that the number of studies are still fairly small. Studies should be conducted in different settings to show consistency and focus on the whole range of pregnancy outcomes including miscarriage, fetal growth, length of gestation, and congenital malformations. Particularly, studies at lower levels of noise are needed, and the shape of the exposure response, including the possibility for thresholds should be evaluated.

Special attention should be paid to the exposure assessment and potential confounders, especially socioeconomic status and air pollution. The exposure assessment should not only include modeled data, but also measurements, noise perception and take into account behavior, timing of exposure and building characteristics such as its acoustic properties (e.g., double-glazed windows, noise insulation, etc.), bedroom orientation (towards or away from road), floor, etc.). It is important to obtain information on potential confounders including other environmental data such as air pollution, which may occur often simultaneously in the case of traffic noise and can affect pregnancy outcomes. The accuracy level of assessment of confounders including air pollution should be at the same level of accuracy as the noise assessment to be able to make sensible comparisons of risk estimates. Furthermore, work is needed on the mechanisms explaining the possible relationships. New OMICs technologies provide great opportunities to provide new insights into the mechanisms underlying the effects of noise [35].

## 7. Conclusions

We found evidence for associations between aircraft noise and preterm birth, low birth weight and congenital anomalies; however, the quality of this evidence could be considered as very low. We also found evidence for an association between road traffic noise and low birth weight, preterm birth and small for gestational age with the quality of evidence being low. Thus, there is a need for further studies with robust exposure assessment, including other confounding factors, such as socioeconomic status and air pollution, and evaluating role of potential modifiers such as noise sensitivity.

## Figures and Tables

**Table 1:** WHO inclusion and exclusion criteria.

	Inclusion Criteria	Exclusion Criteria
Population: general population in settings (hospitals, residences, public venues, educational facilities)	Studies including members of the general population Studies including specific segments of the population particularly at risk, such as pregnant women and newborns Studies including participants exposed to environmental noise; participants exposed to environmental and occupational combined; participants exposed to environmental noise and other environmental factors	Does not meet inclusion criteria Studies including participants exposed to noise in occupational settings not relevant with combined exposure to environmental noise
Exposure: exposure to high levels of environmental noise from various noise sources	Noise exposure levels either measured or calculated and expressed in decibel values. noise levels were representative for the exposure assessment of the study participants (for most observational studies, this would be the dwelling location or home) Calculated levels for transportation noise (road, rail, air) based on traffic data reflecting the use of roads, railway lines and in- and outbound flight routes at airports	Does not meet inclusion criteria Studies using hearing loss or defective hearing as a proxy for (previous) noise exposure Surveys with subjective noise exposure assessment, on the basis of subjective ratings
Comparator: no noise exposure or lower levels of noise exposure	Study have comparator group (corresponding to no exposure or lower level exposure)	Does not meet inclusion criteria
Confounding: adjusted for confounding factors	No inclusion criteria applied; however, for each study, we will assess which possible confounders have been taken into account	No exclusion criteria applied
Outcome: assessment of outcome	Data about outcomes taken from medical records or interview using a known scale or validated assessment method Self-reported data about outcome taken from questionnaire	Does not meet inclusion criteria

**Table 2:** Summary of epidemiological studies on environmental aircraft noise exposure and birth outcomes (ordered by year of publication).

Author, Year	Country	Study Design	Sample Size	Exposure Assessment	Outcome	Confounding Factors	Potential for Bias	Effect Size	Quality Score
Preterm birth and Birth weight									
Ando and Hattori, 1973 [10]	Japan	Case-control study	713	Objective assessment, aircraft noise, ECPNL (dB)	LBW (<2500 g)	Gender, maternal age, occupation, parity	High	Higher rate of LBW in noisy area above 75 dBA	8
Knipschild et al., 1981 [11]	Netherlands	Case-control study	1840	Objective assessment, aircraft noise, 3 subgroups Ldn < 65 dBA, Ldn 65–70 dBA, Ldn > 70 dBA	LBW	Gender, parental income	High	18% LBW in low noise exposed group, 24% LBW in high noise exposed group, 29% in noise exposed above 70 dBA Dose response relationship between aircraft noise and LBW	8
Schell, 1981 [12]	USA	Cross-sectional study	115	Objective assessment, aircraft noise, SEL = 75–100 dBA	Birth weight Gestational length	Maternal age, obstetric history, parental weight and height, education, smoking, family income	High	r = −0.04, *p* = 0.76 males r = −0.22, *p* = 0.014 females r = −0.18, *p* = 0.16 males r = −0.38, *p* = 0.008 females	11
Matsui et al., 2003 [13]	Japan	Survey	160,460 births	Objective assessment, aircraft noise, WECPNL (dB) Control group <75 dBA Exposed subgroups 75–80 dBA, 81–85 dBA, >85 dBA	LBW (<2500 g) Preterm birth (<37 weeks)	Gender, maternal age, socio-economic status, live birth order No adjustment for smoking	High	OR = 1.32 (95% CI 1.18–1.48), *p* = 0.0001 in the highest level of exposure compared to control group OR = 1.25 (95% CI 1.1–1.4), *p* = 0.0018 in the highest level of exposure compared to control group	10
Congenital malformations									
Jones and Tauscher, 1978 [14]	USA	Ecological study	225146 births 2105 defects	Above vs. below >90 dBA	Birth defects	Information not provided	High	1185 vs. 737 per 100,000 births *p* < 0.02	8
Edmonds et al., 1979 [15]	USA	Survey	1745 birth defects	Objective assessment, aircraft noise, high noise level exposure above 65 dBA Ldn	17 categories of birth defects	Socioeconomic status, race	High	Non significant differences in rates of birth defects in exposed and non-exposed groups	10

Notes: ECPNL (Equivalent Continuous Perceived Noise level), SEL (Sound Exposure Level), r (correlation coefficient).

**Table 3:** Summary of epidemiological studies on environmental traffic noise exposure and birth outcomes (ordered by year of publication).

Author, Year	Country	Study Design	Sample Size	Exposure Assessment	Outcome	Confounding Factors	Potential for Bias	Effect Size	Quality Score
Wu et al., 1996 [16]	Taiwan	Prospective study	200	Objective and subjective assessment, Leq 24 h of traffic and total noise	LBW	Maternal age, weight gain, gender and gestational age, socioeconomic status	Low	Non-significant correlation between traffic noise exposure and LBW (*p* = 0.17)	13
Gehring et al., 2014 [17]	Canada	Retrospective study of birth records population-based cohort study	68,238 births	Objective, all transportation and road traffic noise modeling	Preterm birth Term LBW Small for gestational age	Gender, ethnicity, parity, family income, education, smoking, air pollution	Low	All road traffic noise (per 6 dB(A) increase OR = 1.02 (95% CI 0.98–1.06) OR = 1.11 (95% CI 1.03–1.19) OR = 1.09 (95% CI 1.06–1.12)	13
Dadvand et al. 2014 [18]	Spain	Retrospective study of birth records population-based cohort study	6438	Objective, traffic noise modeling	Term LBW	Gender, ethnicity, marital status season of conception, parity, education, smoking, BMI, alcohol consumption, air pollution, temperature	Low	RR = 1.03 (95% CI 0.84–1.27) per 6.7 dB(A)	13
Hystadt et al., 2014 [19]	Canada	Retrospective study of birth records population-based cohort study	64,705 births	Objective, all transportation and road traffic noise modeling	Preterm birth Small for gestational age	Gender, ethnicity, parity, family income, education, smoking, air pollution	Low	All road traffic noise (per 6 dB(A) increase OR = 1.02 (95% CI 0.98–1.06) OR = 1.09 (95% CI 1.06–1.12)	13
Hjortebjerg et al. (2016) [20]	Denmark	Cohort study	75,166 live-born singletons born at term	Calculation method for road and railway traffic noise at the residential address	Term birth weight,	Gestational age sex. maternal age at conception, pre-pregnancy BMI, maternal height, parity, season of conception, educational level, disposable income, smoking and alcohol consumption, air pollution.	low	OR: 1.07 (95% CI: 0.94; 1.21) per 10 dB) No associations after full adjustment	13
Arroyo et al. (2016a) [21]	Spain	Ecological time series study	298,705 births	Objective noise measurements from 26 monitoring stations in Madrid Mean Leqd = 64.6 dB(A) Mean Leqn = 59.4 dB(A)	LBW Premature birth	Not considered, air pollution and temperature are controlled variables	high	Transportation noise Leqd (3rd tr) RR = 1.01 (95% CI 1.00–1.02) Leqd (2nd tr) RR = 1.04 (95% CI 1.03–1.05) Leqd (1st tr) RR = 1.03 (95% CI 1.02–1.04) Leqd (2nd tr) RR=1.03 (95%CI 1.02–1.03) Leqn (3rd tr) RR = 1.02 (95%CI 1.01–1.02)	10
Arroyo et al. (2016b) [22]	Spain	Ecological time series study	298,705 births	Objective noise measurements from 26 monitoring stations in Madrid Mean Leqd = 64.6 dB(A) Mean Leqn = 59.4 dB(A)	Very Preterm births (30- < 37 weeks) Extremely preterm births (<30 weeks)	Not considered, air pollution and temperature are controlled variables	high	Transportation noise Leqd (Lag 0) RR = 1.07 (95% CI 1.04–1.10) Leqd (Lag 0) RR = 1.28 (95% CI 1.21–1.36)	10
Diaz et al. (2016) [23]	Spain	Ecological time series study	298,705 births	Measured noise levels from monitoring stations	LBW in non-premature births Very LBW Extremely LBW	Not considered, air pollution and temperature are controlled variables	low	All noise RR = 1.09 (95% CI 0.99–1.19) (*p* < 0.1).	10

Notes: OR (Odds Ratio), CI (Confidence Intervals), Leqd (equivalent diurnal noise (7–23 h), Leqn (equivalent nocturnal noise (23–7 h)). tr (trimester).

## Supplementary Materials

The following are available online at www.mdpi.com/1660-4601/14/10/1252/s1, Table S1: GRADE for the quality of evidence of aircraft noise associated with pre-term birth, Table S2: GRADE for the quality of evidence of aircraft noise associated with low birth weight, Table S3: GRADE for the quality of evidence of aircraft noise associated with congenital malformations, Table S4: GRADE for the quality of evidence of road traffic noise associated with pre-term birth, Table S5: GRADE for the quality of evidence of road traffic noise associated with low birth weight, Table S6: GRADE for the quality of evidence of road traffic noise associated with small for gestational age, Table S7: Assessment of the risk of bias in the individual studies for Table S1, Table S8: Assessment of the risk of bias in the individual studies for Table S2, Table S9: Assessment of the risk of bias in the individual studies for Table S3, Table S10: Assessment of the risk of bias in the individual studies for Table S4, Table S11: Assessment of the risk of bias in the individual studies for Table S5, Table S12: Assessment of the risk of bias in the individual studies for Table S6.
