# Supplementary material for: WHO Environmental Noise Guidelines for the European Region: A Systematic Review on Environmental Noise and Adverse Birth Outcomes
Source: Int J Environ Res Public Health. 2017 Oct 19;14(10):1252. doi: 10.3390/ijerph14101252 (PMC5664753; doi:10.3390/ijerph14101252)
Supplement: Supplementary file 1 [file ijerph-14-01252-s001.pdf]

Supplementary materials

Table S1. GRADE for the quality of evidence of aircraft noise associated with pre-term delivery.

Domains	Criterion	Assessment	Downgrading
**Start Level**		**Cross-sectional and survey**	**Low quality**
1. Study Limitations	Majority of studies low quality	few studies of low quality	**Downgrade one level**
2. Inconsistency	Conflicting results; high I^2	Difficult to judge	**Downgrade one level**
3. Directness	Direct comparison; same PECCO	Good	**No downgrade**
4. Precision	Confidence interval contains 25% harm or benefit	Difficult to judge	**No downgrade**
5. Publication Bias	Funnel plot indicates	No information available	**No downgrade**
6. Dose-response	Significant trend	Difficult to judge	**No upgrade**
7. Magnitude of effect	RR > 2	No	**No upgrade**
8. Confounding adjusted	Effect in spite of confounding working towards the nil	Unclear	**No upgrade**
**Overall Judgment**			**Very low**

Table S2. GRADE for the quality of evidence of aircraft noise associated with low birth weight.

Domains	Criterion	Assessment	Downgrading
**Start Level**		**Case-control studies**	**Low quality**
1. Study Limitations	Majority of studies low quality	Yes	**Downgrade one level**
2. Inconsistency	Conflicting results; high I^2	Similar direction but overall unclear	**Downgrade one level**
3. Directness	Direct comparison; same PECCO	Yes direct evidence	**No downgrade**
4. Precision	Confidence interval contains 25% harm or benefit	Only for one study clearly	**Downgrade one level**
5. Publication Bias	Funnel plot indicates	Not able to assess	**No downgrade**
6. Dose-response	Significant trend	Only for one study clearly	**No upgrade**
7. Magnitude of effect	RR > 2	No	**No upgrade**
8. Confounding adjusted	Effect in spite of confounding working towards the nil	Not fully adjusted	**No upgrade**
**Overall Judgment**			**Very low**

Table S3. GRADE for the quality of evidence of aircraft noise associated with congenital malformations.

Domains	Criterion	Assessment	Downgrading
**Start Level**		**Ecological study and survey**	**Low quality**
1. Study Limitations	Majority of studies low quality	Yes	**Downgrade one level**
2. Inconsistency	Conflicting results; high I^2	yes	**Downgrade one level**
3. Directness	Direct comparison; same PECCO	Yes	**No downgrade**
4. Precision	Confidence interval contains 25% harm	No	**Downgrade one level**

	or benefit		
5. Publication Bias	Funnel plot indicates	No	**No downgrade**
6. Dose-response	Significant trend	No	**No upgrade**
7. Magnitude of effect	RR > 2	No	**No upgrade**
8. Confounding adjusted	Effect in spite of confounding working towards the nil	No	**No upgrade**
**Overall Judgement**			**Very low**

Table S4. GRADE for the quality of evidence of road traffic noise associated with pre-term delivery

**Domains**	**Criterion**	**Assessment**	**Downgrading**
**Start Level**		**Cohort study**	**High quality**
1. Study Limitations	Majority of studies low quality	Only one study of good quality	**No downgrade**
2. Inconsistency	Conflicting results; high I^2	Only one study	**Downgrade one level**
3. Directness	Direct comparison; same PECCO	Yes	**No downgrade**
4. Precision	Confidence interval contains 25% harm or benefit	No	**Downgrade one level**
5. Publication Bias	Funnel plot indicates	Not able to assess	**No downgrade**
6. Dose-response	Significant trend	No	**No upgrade**
7. Magnitude of effect	RR > 2	No	**No upgrade**
8. Confounding adjusted	Effect in spite of confounding working towards the nil	No	**No upgrade**
**Overall Judgment**			**Low Quality**

Table S5. GRADE for the quality of evidence of road traffic noise associated with low birth weight.

Domains	Criterion	Assessment	Downgrading
**Start Level**		**Cohort studies**	**High quality**
1. Study Limitations	Majority of studies low quality	No, all low risk of bias	**No downgrade**
2. Inconsistency	Conflicting results; high I^2	Inconsistent results	**Downgrade one level**
3. Directness	Direct comparison; same PECCO	yes	**No downgrade**
4. Precision	Confidence interval contains 25% harm or benefit	Not precise	**Downgrade one level**
5. Publication Bias	Funnel plot indicates	Not able to assess	**No downgrade**
6. Dose-response	Significant trend	Not in all studies	**No upgrade**
7. Magnitude of effect	RR > 2	No	**No upgrade**
8. Confounding adjusted	Effect in spite of confounding working towards the nil	Most confounders are connected to noise levels and outcomes would increase the risk	**No upgrade**
**Overall Judgment**			**Low Quality**

Table S6. GRADE for the quality of evidence of road traffic noise associated with small for gestational age.

**Domains**	**Criterion**	**Assessment**	**Downgrading**
**Start Level**		**Cohort study**	**High quality**
1. Study Limitations	Majority of studies low quality	No	**No downgrade**
2. Inconsistency	Conflicting results; high I^2	Two studies based on the same subjects and exposure assessment	**Downgrade one level**
3. Directness	Direct comparison; same PECCO	yes	**No downgrade**
4. Precision	Confidence interval contains 25% harm or benefit	No	**Downgrade one level**
5. Publication Bias	Funnel plot indicates	Not able to assess	**No downgrade**
6. Dose-response	Significant trend	Not able to assess	**No upgrade**
7. Magnitude of effect	RR > 2	No	**No upgrade**
8. Confounding adjusted	Effect in spite of confounding working towards the nil	Yes	**No upgrade**
**Overall Judgment**			**Low**

Table S7 Assessment of the risk of bias in the individual studies for Table S1

Ref.	year	Bias due to selection of participants	Information bias due to exposure assessment	Outcome bias I	Outcome bias II	Bias due to confounding	Count of columns with low risk of bias	Total risk of bias
Schell [11]	1981	high	low	high	high	low	2	high
Matsui et al [13]	2007	low	high	low	high	high	2	high

Table S8 Assessment of the risk of bias in the individual studies for Table S2

Ref.	year	Bias due to selection of participants	Information bias due to exposure assessment	Outcome bias I	Outcome bias II	Bias due to confounding	Count of columns with low risk of bias	Total risk of bias
Ando and Hattori [10]	1973	high	low	high	high	high	1	high
Schell [11]	1981	high	low	high	high	low	2	high
Knipschild et al [12]	1981	low	low	high	high	high	2	high
Matsui et al [13]	2007	low	high	low	high	high	1	high

Table S9 Assessment of the risk of bias in the individual studies for Table S3

Ref.	year	Bias due to selection of participants	Information bias due to exposure assessment	Outcome bias I	Outcome bias II	Bias due to confounding	Count of columns with low risk of bias	Total risk of bias
Jones and Tauscher [14]	1978	low	high	low	high	high	2	high

Edmonds et al [15]	1979	low	high	low	high	high	2	high

Table S10 Assessment of the risk of bias in the individual studies for Table S4

		Bias due to selection of participants	Information bias due to exposure assessment	Outcome bias I	Outcome bias II	Bias due to confounding	Count of columns with low risk of bias	Total risk of bias
Gehring et al [17]	2014	low	high	low	low	low	4	low
Hystadt et al [19]	2014	low	high	low	low	low	4	low
Arroyo et al [22]	2016a	low	low	high	high	high	2	high
Arroyo et al [21]	2016b	low	low	high	high	high	2	high

Table S11 Assessment of the risk of bias in the individual studies for Table S5

		**Bias due to selection of participants**	**Information bias due to exposure assessment**	**Outcome bias I**	**Outcome bias II**	**Bias due to confounding**	**Count of columns with low risk of bias**	**Total risk of bias**
Wu et al [16]	1996	low	low	low	low	high	4	low
Gehring et al [17]	2014	low	high	low	low	low	4	low
Dadvand et al [18]	2014	low	high	low	low	low	4	low
Hjortebjerg et al [20]	2016	low	low	low	high	low	4	low
Arroyo et al [21]	2016b	low	low	high	high	high	2	high

Diaz et al [23]	2016	low	low	high	high	high	2	high

Table S12 Assessment of the risk of bias in the individual studies for Table S6

		Bias due to selection of participants	Information bias due to exposure assessment	Outcome bias I	Outcome bias II	Bias due to confounding	Count of columns with low risk of bias	Total risk of bias
Gehring et al [17]	2014	low	high	low	low	low	4	low
Hystadt et al [19]	2014	low	high	low	low	low	4	low
